# Latencies longer than 3.5 ms after vagus nerve stimulation does not exclude a nonrecurrent inferior laryngeal nerve

**DOI:** 10.1186/1471-2482-14-61

**Published:** 2014-08-28

**Authors:** Michael Brauckhoff, Helle Naterstad, Katrin Brauckhoff, Martin Biermann, Turid Aas

**Affiliations:** 1 Department of Surgery, Haukeland University Hospital, Bergen, Norway; 2 Department of Clinical Science, University of Bergen, Bergen, Norway; 3 Department of Radiology, NM/PET-Centre, Haukeland University Hospital, Bergen, Norway; 4 Department of Clinical Medicine, University of Bergen, Bergen, Norway; 5 Haukeland University Hospital Bergen, Jonas Lies vei 65, 5021 Bergen, Norway

**Keywords:** Nonrecurrent laryngeal nerve, Intraoperative neurostimulation, Latency, Nerve conduction velocity

## Abstract

**Background:**

It has recently been reported that a signal latency shorter than 3.5 ms after electrical stimulation of the vagus nerve signify a nonrecurrent course of the inferior laryngeal nerve. We present a patient with an ascending nonrecurrent inferior laryngeal nerve. In this patient, the stimulation latency was longer than 3.5 ms.

**Case presentation:**

A 74-years old female underwent redo surgery due to a right-sided recurrent nodular goitre. The signal latency on electrical stimulation of the vagus nerve at the level of the carotid artery bifurcation was 3.75 ms. Further dissection revealed a nonrecurrent but ascending course of the inferior laryngeal nerve. Caused by the recurrent goitre, the nerve was elongated to about 10 cm resulting in this long latency.

**Conclusion:**

This case demonstrates that the formerly proposed “3.5 ms rule” for identifying a nonrecurrent course of the inferior laryngeal nerve has exceptions. A longer latency does not necessarily exclude a nonrecurrent laryngeal nerve.

## Background

It has recently been reported that a signal latency shorter than 3.5 ms after electrical stimulation of the vagus nerve (VN) signify a nonrecurrent course of the inferior laryngeal nerve (ILN) [1]. In intraoperative neuromonitoring (ION), signal latency mainly depends on two factors: (a) the distance between the stimulation site and the location of signal recording (in case of ION the intralaryngeal muscles), and (b) the nerve conduction velocity (NCV), which is about 75 m/s in humans [2].

A nonrecurrent inferior laryngeal nerve (NRILN) branches from the VN not in the mediastinum but in the neck and turns directly to the larynx. This anatomical variant can be found in about 0.5% [3-5]. For embryological reasons, a NRILN occurs almost exclusively on the right side. The NRILN is associated with an aberrant right subclavian artery that branches from the left side of the aortic arch [6]. Whereas the ILN normally runs around the right subclavian artery as recurrent laryngeal nerve (RLN), a NRILN has a more or less horizontal course in the neck usually leaving the VN at the level of the larynx. The consequent difference in length between the RLN and the NRILN of about 10–15 cm results in shorter signal latencies when stimulating the VN. The median signal latency in RLN and NRILN is 4.6 ms vs. 2.7 ms (p < .001), respectively [1]. Based on these findings, a cut off of 3.5 ms has been defined to distinguish the usual recurrent course from the atypical nonrecurrent course of the ILN [1].

This “3.5 ms rule” has, however, limitations as the present case will demonstrate.

## Case presentation

**Figure 1:**
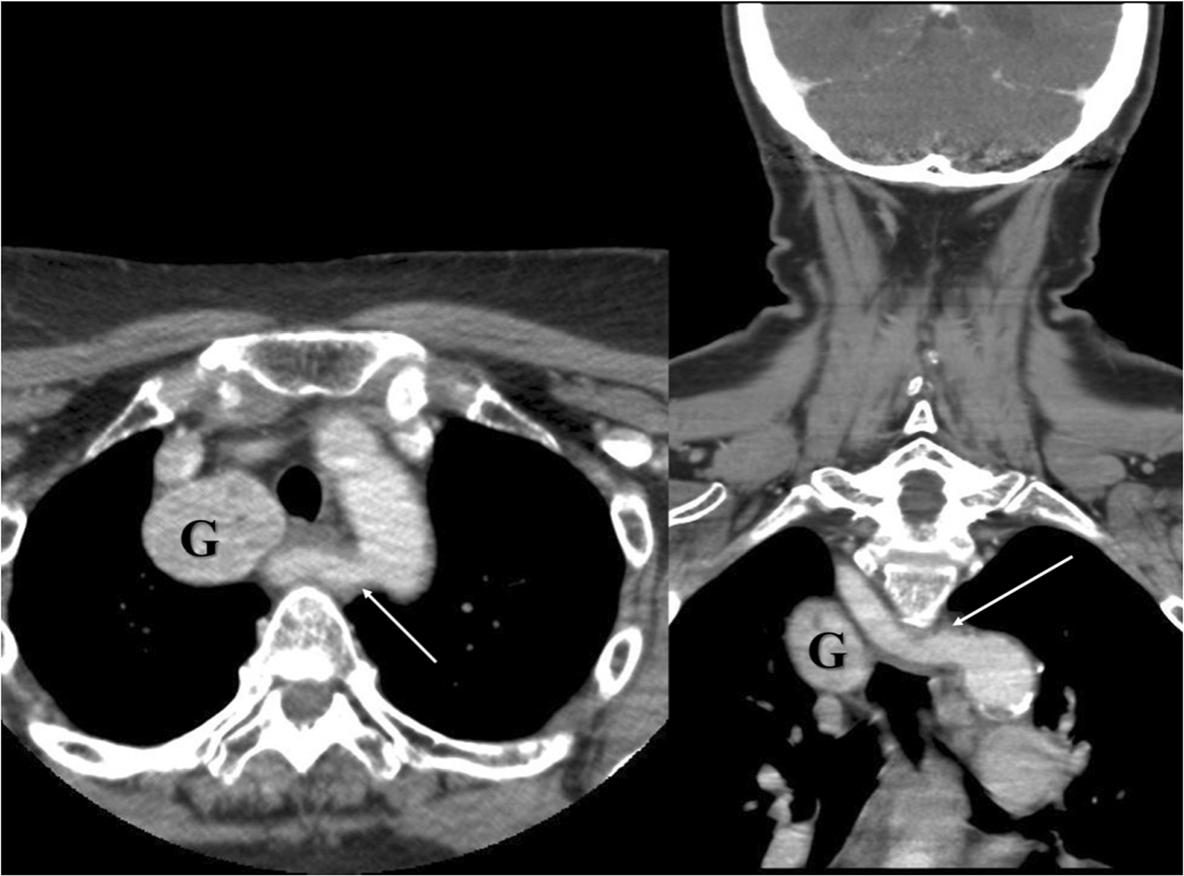
Preoperative CT scan indicating a mediastinal goitre (G) and an aberrant right subclavian artery (white arrow).

**Figure 2:**
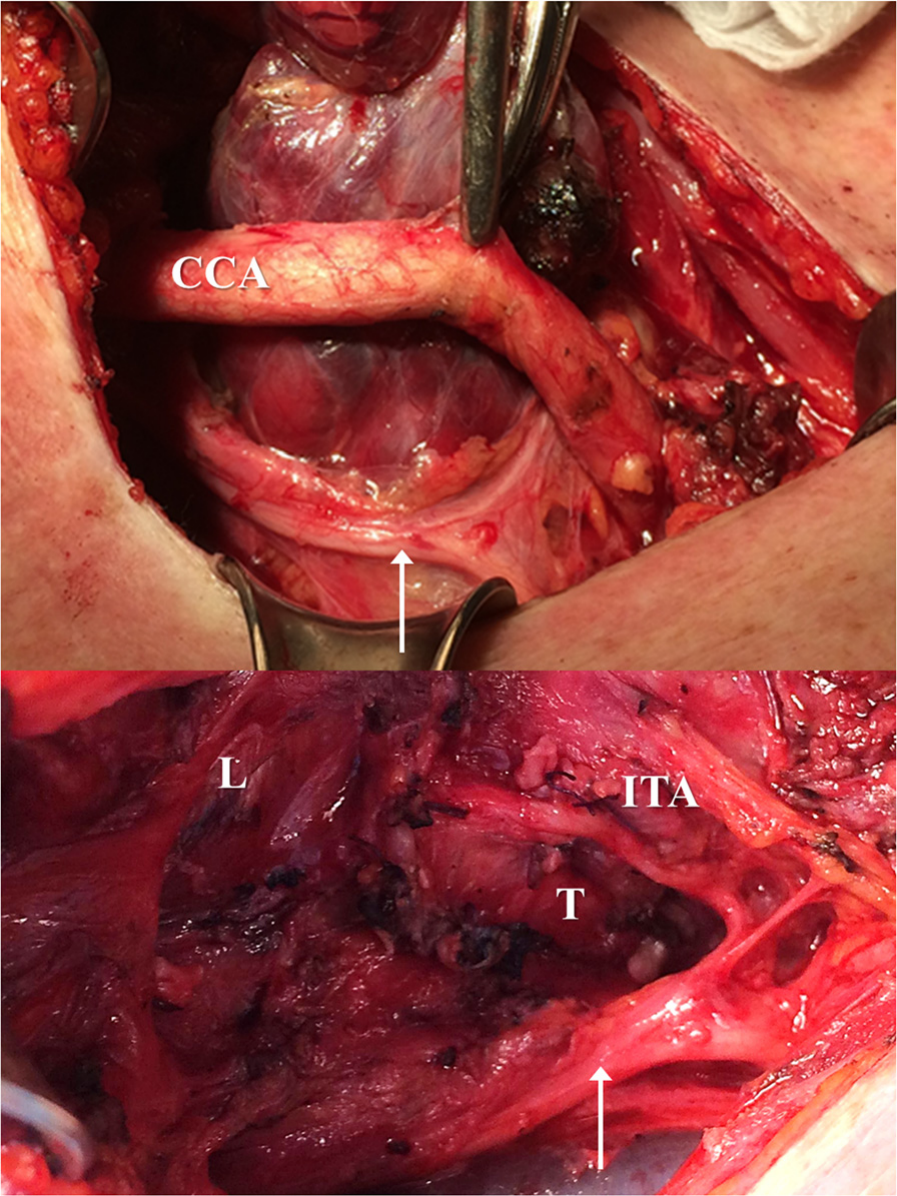
**Anatomy of the elongated NRILN with deep separation from the VN (white arrow) and ascending course before and after hemithyroidectomy.** CCA, common carotid artery. ITA, Inferior thyroid artery (ligated). L, Larynx. T, Trachea.

**Figure 3:**
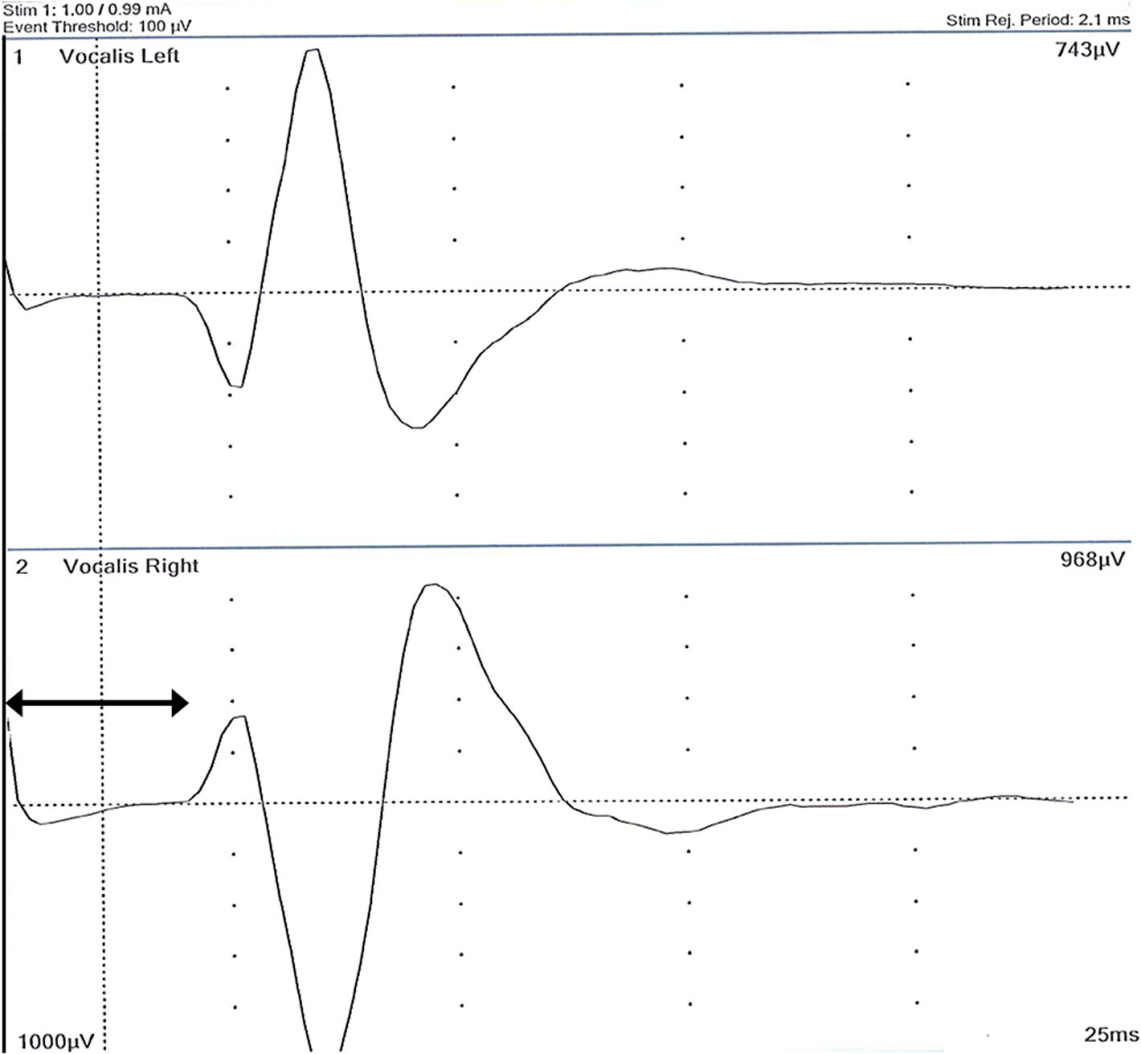
EMG of the vagus nerve stimulated at the level of the carotid artery bifurcation with a signal latency of 3.75 ms.

A 74 year old Caucasian female had undergone subtotal right-sided lobectomy due to nodular goitre in 1974. She now presented with dyspnoea due to tracheal compression by a large recurrent right-sided goitre of about 10.5 × 4.5 × 3.2 cm (calculated volume 75 ml). Preoperative computed tomography (CT) showed mediastinal goitre and an aberrant right subclavian artery (Figure 1). Based on this finding, a nonrecurrent course of the ILN was suggested. Surgery was performed using ION as formerly described [1].Surprisingly, VN stimulation at the level of the inferior thyroid artery produced a normal electromyography (EMG) signal with a latency of about 3.3 ms which under normal circumstances would rather indicate a RLN. Since, based on the preoperative CT scan, however, a nonrecurrent course of the nerve had to be expected, a more extensive dissection of the VN was performed. The separation of the ILN from the VN was found deeper in the neck (Figure 2) indicating nonetheless a NRILN. The NRLIN was elongated by the expansively growing recurrent goitre with a deep separation from the cervical VN resulting in an ascending course to the larynx and a length of about 10 cm (Figure 2). ION repeated at the level of the carotid artery bifurcation gave a latency of 3.75 ms (Figure 3).

## Discussion

Since electrical VN stimulation distal to the separation of the ILN will not produce any EMG response, a NRILN can be identified by negative EMG response after distal but positive EMG response after proximal VN stimulation before the nerve is morphologically identified [5]. The concept of “distal VN stimulation first” introduced by our group [5] has recently been confirmed [7].

Compared to the usual course of the RLN that runs around the right subclavian artery, a NRLIN is about 10–15 cm shorter. The NCV of the RLN is about 75–80 m/s in humans [2]. When stimulating the VN before the laryngeal fascicles leave the VN, the difference in length between NRILN and RLN thus results in a shortening of the latency of the EMG signal by 1.5-2 ms [1].

It has recently been reported by our group that the median latency after VN stimulation in adults with a NRILN is 2.7 ms but 4.6 ms in normal RLN (p < .001). A cut-off of 3.5 ms had positive and negative predictive values of 100% and 97%, respectively. We concluded that “proximal VN stimulation only” might replace the mentioned “distal VN stimulation first” approach [1].

Regarding the latency of the EMG signal, however, the site of VN stimulation is crucial. Assuming a NCV of 75 m/s, a 3.75 cm distance between two stimulation sites will result in a 0.5 ms difference in latency. The latencies for right-sided VN stimulation reported in our above mentioned study were based on a high VN stimulation site at the level of the carotid artery bifurcation [1] and are therefore longer than in a similar study that reported a median latency after right VN stimulation of 3.91 (10th/90th percentile 3.13/4.69) ms but did not define the precise site of VN stimulation [8]. The distance between the carotid bifurcation and the subclavian artery is obviously variable in humans, which potentially limits the “3.5 ms rule”. However, there is a scarcity of other applicable landmarks in the neck. The carotid bifurcation has the advantage that it is high enough in the neck allowing the recruitment of ILN fascicles by VN stimulation independent of the course of the ILN (descending, transverse, or ascending NRILN, or usual RLN respectively). At the same time, it is the highest and most reliable landmark in the neck that can be reached by a conventional Kocher incision.

Based on previous reports describing descending and ascending courses [9], potential limitations of this “3.5 ms rule” have to be considered. An ascending course of the NRILN might result in longer latency than the usual horizontal NRILN [1]. No such cases, however, have so far been documented electrophysiologically.

## Conclusion

The present case demonstrates that the formerly proposed “3.5 ms rule” may indeed have exceptions. Particularly in large goitres with possibly elongation of the nerve, a longer latency does not necessarily exclude a NRILN. Although the main conclusion of our previous report is still unrefuted - *Latencies Shorter than 3.5 ms After Vagus Nerve Stimulation Signify a Nonrecurrent Inferior Laryngeal Nerve*[1] - , the opposite proposition that longer latencies exclude a NRILN is not necessarily true.

## Consent

The patient gave written informed consent to the publication of this case report and accompanying images. A copy of the written consent is available for review by the Editor of the journal.

## Abbreviations

EMG: Electromyography; ILN: Inferior laryngeal nerve; ION: Intraoperative neuromonitoring; NCV: Nerve conduction velocity; NRILN: Nonrecurrent inferior laryngeal nerve; RLN: Recurrent inferior laryngeal nerve; VN: Vagus nerve.

## Competing interests

The authors declare that they have no competing interests.

## Authors’ contributions

MBrau has collected the data and drafted the manuscript. HN has collected the data and drafted the manuscript. KB has collected the data and drafted the manuscript. MBier was helping collecting data and drafting the manuscript. TA was involved in drafting the manuscript. All authors read and approved the final manuscript.

## Pre-publication history

The pre-publication history for this paper can be accessed here:

http://www.biomedcentral.com/1471-2482/14/61/prepub
